# JC Polyomavirus Uses Extracellular Vesicles To Infect Target Cells

**DOI:** 10.1128/mBio.00379-19

**Published:** 2019-04-09

**Authors:** Jenna Morris-Love, Gretchen V. Gee, Bethany A. O’Hara, Benedetta Assetta, Abigail L. Atkinson, Aisling S. Dugan, Sheila A. Haley, Walter J. Atwood

**Affiliations:** a Department of Molecular Biology, Cell Biology, and Biochemistry, Brown University, Providence, Rhode Island, USA; b Graduate Program in Pathobiology, Brown University, Providence, Rhode Island, USA; c Department of Natural Sciences, Assumption College, Worcester, Massachusetts, USA; Baylor College of Medicine; University of Michigan; Fred Hutchison Cancer Research Center

**Keywords:** progressive multifocal leukoencephalopathy, central nervous system infections, extracellular vesicle, polyomavirus, virus receptor

## Abstract

The endemic human JC polyomavirus (JCPyV) causes progressive multifocal leukoencephalopathy in immune-suppressed patients. The mechanisms of virus infection *in vivo* are not understood because the major target cells for virus in the brain do not express virus receptors and do not bind virus. We found that JCPyV associates with extracellular vesicles (EVs) and can infect target cells independently of virus receptors. Virus particles were found packaged inside extracellular vesicles and attached to the outer side of vesicles. Anti-JCPyV antisera reduced infection by purified virus but had no effect on infection by EV-associated virus. Treatment of cells with the receptor-destroying enzyme neuraminidase inhibited infection with purified virus but did not inhibit infection by EV-associated virus. Mutant pseudoviruses defective in sialic acid receptor binding could not transduce cells as purified pseudovirions but could do so when associated with EVs. This alternative mechanism of infection likely plays a critical role in the dissemination and spread of JCPyV both to and within the central nervous system.

## Abstract (precis)

JC polyomavirus (JCPyV) is a ubiquitous human pathogen that causes progressive multifocal leukoencephalopathy (PML), a severe and often fatal neurodegenerative disease in immunocompromised or immunomodulated patients. The mechanisms responsible for initiating infection in susceptible cells are not completely known. The major attachment receptor for the virus, lactoseries tetrasaccharide c (LSTc), is paradoxically not expressed on oligodendrocytes or astrocytes in human brain, and virus does not bind to these cells. Because these are the major cell types targeted by the virus in the brain, we hypothesized that alternative mechanisms of infection must be responsible. Here we provide evidence that JCPyV is packaged in extracellular vesicles from infected cells. Infection of target cells by vesicle-associated virus is not dependent on LSTc and is not neutralized by antisera directed against the virus. This is the first demonstration of a polyomavirus using extracellular vesicles as a means of transmission.

## INTRODUCTION

JC polyomavirus (JCPyV) is a small nonenveloped virus that establishes persistent infection in over half the world’s population. In immunosuppressed or immunomodulated patients, JCPyV spreads to the central nervous system (CNS), where infection of oligodendrocytes leads to a rapidly progressing and severely debilitating demyelinating disease known as progressive multifocal leukoencephalopathy (PML) (1–5). JCPyV enters cells by a two-step mechanism in which the VP1 major viral capsid protein specifically attaches to the host cell via the sialic acid moiety of lactoseries tetrasaccharide C (LSTc) and then binds one of the members of the 5-hydroxytryptamine (5HT2) family of serotonin receptors for internalization (6–9). Internalization of receptor and virus occurs by clathrin-mediated endocytosis and is followed by viral trafficking to the endoplasmic reticulum and nucleus for replication (8, 10–12). JCPyV strains found in the CNS of PML patients accumulate mutations in the LSTc-binding pocket of VP1, rendering these viruses incapable of binding the sialic acid moiety of LSTc (6, 13, 14). Confounding this paradox is the finding that oligodendrocytes and astrocytes, the main targets of JCPyV infection in the CNS, do not express LSTc and do not bind virus (15, 16). We therefore hypothesized that a receptor-independent mechanism of infection was responsible for virus spread within the CNS.

Several viruses use extracellular vesicles (EVs) as delivery systems, including HIV-1, hepatitis A virus, some herpesviruses, and as determined most recently, rotaviruses and noroviruses (17–22). In the case of noroviruses and rotaviruses, extracellular vesicles are significantly more infectious than individual viral particles and likely play an important role in fecal-oral transmission and pathogenesis (22). Extracellular vesicles also carry an abundance of regulatory molecules, including microRNAs, that influence the microenvironment of target tissues (23–25).

Here, we show for the first time that the human polyomavirus JCPyV associates with and is packaged in extracellular vesicles. The virus-containing vesicles are highly infectious, and the infection is not neutralized by antiviral antisera or inhibited by treatment of cells with neuraminidase. This mechanism is likely responsible for virus infection of oligodendrocytes and astrocytes, both of which lack the sialic acid attachment receptor for the virus and fail to bind virus *in vivo*. This mechanism is also likely responsible for the accumulation and spread of sialic acid binding mutant viruses in the cerebral spinal fluid (CSF) and brain parenchyma of patients with PML.

## RESULTS

### JC polyomavirus associates with extracellular vesicles and infects target cells.

**FIG 1:**
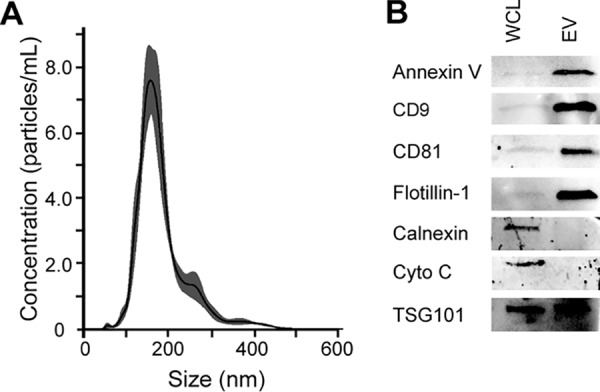
JCPyV-infected SVG-A cells produce extracellular vesicles. (A) Extracellular vesicles were purified from infected SVG-A cells by differential centrifugation. The final EV pellet was resuspended in PBS and diluted 1:100 in PBS for nanoparticle tracking analysis. Five videos were recorded and used for analysis, with outputs of concentration in particles per milliliter and size in nanometers. Data are representative of averages. (B) Extracellular vesicles (EV) were purified from cell supernatants, lysed, and resolved on 12% SDS-PAGE (EV). Whole-cell lysates (WCL) were also run in parallel. The blots were probed with antibodies against annexin V, CD9, CD81, flotillin-1, calnexin, cytochrome *c*, and TSG101.

**FIG 2:**
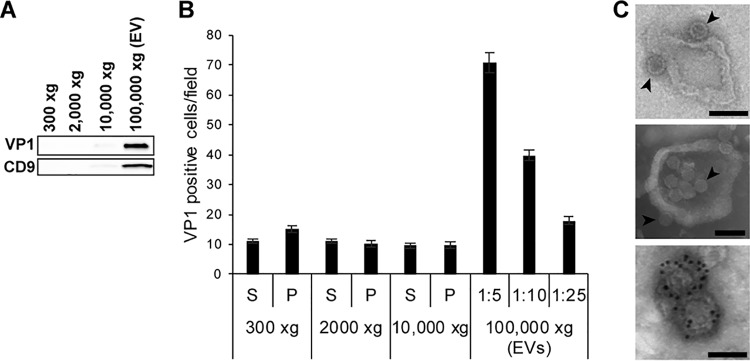
Extracellular vesicles contain JCPyV and are infectious. (A) Pellets from each step during the ultracentrifugation purification of EVs were resolved on SDS-PAGE, blotted to a nitrocellulose membrane, and probed with antibody to the major viral capsid protein VP1 and with the EV marker CD9 as indicated. (B) Supernatants and pellets from each step during the ultracentrifugation purification of EVs were used to challenge SVG-A cells. Infection was scored at 5 days postinfection by staining the SVG-A cells with an antibody to the VP1 viral protein. (C) Electron micrographs of purified EV showing virus (black arrowheads) attached to the outer side of vesicles (top panel) and enclosed within some vesicles (middle panel). The EVs were positive for CD81 using immunogold labeled secondary antibody (bottom panel). Scale bars, 100 nM.

To determine whether JCPyV uses extracellular vesicles (EVs) to infect cells, EVs were first purified from JCPyV-infected SVG-A cells by differential ultracentrifugation and subjected to nanoparticle tracking analysis (NTA) for determination of concentrations and size distributions. We found that the SVG-A cells consistently produced between 1 × 10^8^ and 1 × 10^11^ particles/ml with an average size of between 150 to 200 nm (Fig. 1A). Western blot analysis of EVs derived from infected SVG-A cells demonstrated an enrichment of known EV markers, including the tetraspanins CD9 and CD81, flotillin-1, annexin-V, and tumor-susceptibility gene 101 (TSG-101), and showed that they were negative for endoplasmic reticulum marker calnexin and mitochondrial marker cytochrome *c* (Fig. 1B) (26–29). The EVs derived from JCPyV-infected cells were also positive for VP1, the major capsid protein of JCPyV (Fig. 2A). To determine whether infectious virus was present in the EVs, supernatants and pellets from each ultracentrifugation step were used to infect naive SVG-A cells. Five days after challenge, the cells were scored for virus infection by immunofluorescence analysis (IFA) using an antibody against VP1. The extracellular vesicle fraction found in the pellet from the ultracentrifugation performed at 100,000 × *g* had the greatest level of infection, and infection occurred in a dose-dependent manner (Fig. 2B). Transmission electron microscopy (TEM) demonstrated that virus could be attached to EVs or enclosed inside EVs, and immunogold electron microscopy (IEM) showed that these EVs were positive for CD81 (Fig. 2C). EVs were also subjected to an iodixanol stepwise gradient (OptiPrep), and 22 fractions (200 µl) were collected and tested for density and infectivity and for the presence of virus by TEM. Infectious EVs were found in a peak between 1.06 and 1.11 g/ml, which is consistent with membrane association (18, 30), whereas free virus has a buoyant density of 1.20 g/ml (31, 32). Electron micrographs corresponding to the infectious EV peak are shown in Fig. 2C (top two panels).

### JC polyomavirus-associated extracellular vesicles are not inhibited by JCPyV-specific antisera.

**FIG 3:**
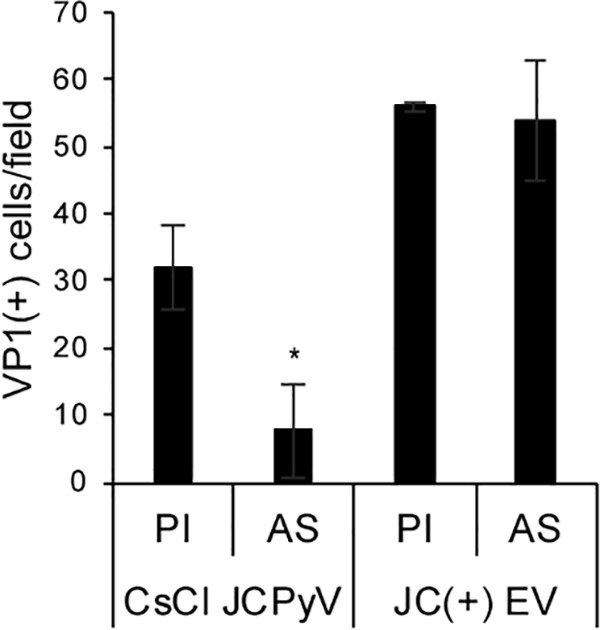
Extracellular vesicle-associated virus is resistant to antibody-mediated neutralization. Polyclonal antisera directed at JCPyV (AS) or preimmune sera (PI) were incubated with cesium chloride-purified JCPyV (CsCl JCPyV) or with extracellular vesicles purified from JCPyV-infected cells (JC(+) EV). Cells were then challenged, and infection was measured by IFA with antibodies against VP1. *, *P* ≤ 0.05.

Because virus was found attached to the outer side of some vesicles, we asked whether the infectivity of the EV-associated virus could be inhibited by neutralizing antisera against JCPyV (33). Purified virus or JCPyV-positive EVs were pretreated with JCPyV-specific antisera (AS) or preimmune sera (PI) and then used to infect naive SVG-A cells. At 5 days postinfection (dpi), cells were fixed and evaluated for infection by immunofluorescence analysis (IFA) for VP1 positive cells. Infection with JCPyV-positive EV was unaffected by the anti-JCPyV antisera, whereas purified JCPyV was significantly inhibited by the antisera (Fig. 3).

### JC polyomavirus-associated extracellular vesicles infect cells in a receptor-independent manner.

**FIG 4:**
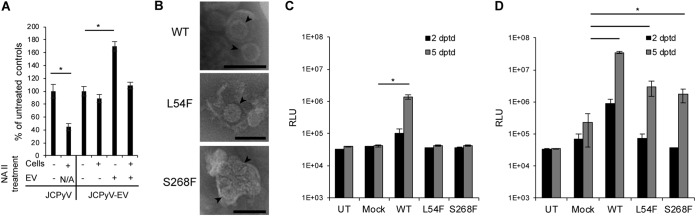
Transmission of virus to naive cells in extracellular vesicles is independent of the virus attachment receptor. (A) SVG-A cells or EV derived from JCPyV-infected SVG-A cells were treated with neuraminidase type II (NA II) as indicated. SVG-A cells were then challenged with purified JCPyV or with extracellular vesicles containing JCPyV (JCPyV-EVs). Infection was measured by staining cells with antibody against VP1. N/A, not applicable. (B) TEM of wild-type (WT) JC pseudovirus (JCPsV-EV) and sialic acid (LSTc) binding pocket mutant pseudoviruses (L54F and S268S) associated with extracellular vesicles. Pseudoviruses are marked with black arrowheads. (C) SVG-A cells were challenged with cesium chloride-purified PsV containing wild-type VP1 (WT) or each of the sialic acid binding pocket mutants of VP1 (L54F and S268F). Transduction was measured by luciferase assay, and the results were compared to the levels determined for the untransduced controls (UT) and mock transductions (lacking the plasmids expressing VP1, VP2, and VP3) at 2 and 5 days posttransduction (dptd). RLU, relative luciferase units. (D) SVG-A cells were challenged with EV containing wild-type or sialic acid pocket mutant pseudoviruses. Transduction was measured by luciferase assay, and the results were compared to the levels determined for the untransduced controls (UT) and mock transductions at 2 and 5 days dptd. *, *P* ≤ 0.05. Scale bars, 100 nM.

To determine whether this mechanism of infection was dependent on the known virus attachment receptor LSTc, we treated cells or extracellular vesicles or both with concentrations of neuraminidase that would remove the major receptor-type sialic acid found on LSTc from the membranes. Treatment of cells with neuraminidase inhibited infection by purified virus but did not inhibit infection by extracellular vesicle-associated virus (Fig. 4A). Treatment of the extracellular vesicles with neuraminidase enhanced infection, and the results of treatment of both the extracellular vesicles and the cells were similar to those seen after treating the cells alone (Fig. 4A). We also analyzed JC pseudoviruses containing wild-type VP1 or VP1 harboring the sialic acid and LSTc binding mutations L54F and S268F. Wild-type and mutant strains were purified as pseudovirions or isolated in extracellular vesicles (Fig. 4B). Pseudoviruses harboring these mutations could not transduce cells as purified pseudovirions (Fig. 4C) but could transduce the cells when associated with extracellular vesicles (Fig. 4D). These data clearly demonstrate that infection of cells by extracellular vesicle-associated virus is independent of sialic acid and LSTc.

**FIG 5:**
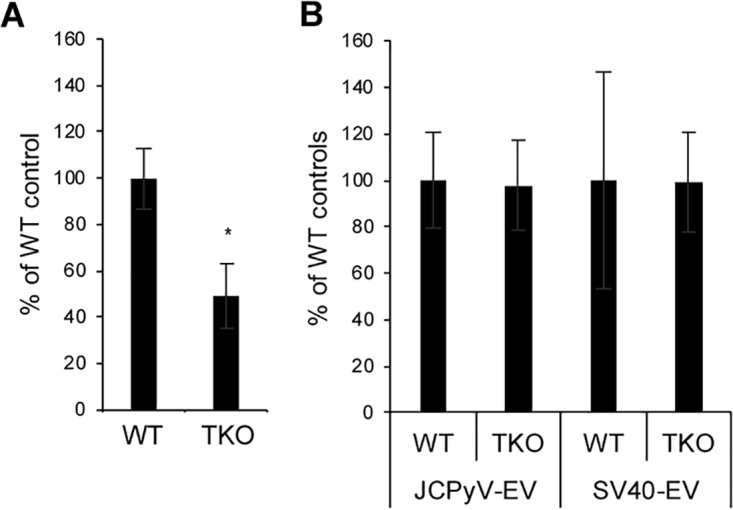
Transmission of virus to naive cells by EV is independent of the virus entry receptors. (A) Infection of wild-type (WT) or 5HT2R genetic triple-knockout SVG-A cells (TKO) with purified virus. (B) Infection of the same cells using EV-associated JCPyV or simian virus 40 (SV40). Wild-type values were set to 100% for comparison. *, *P* ≤ 0.05.

Because infection by purified virus requires the presence of one of three isoforms of the 5HT2 receptor, we asked if the same would be true of EV-mediated infection. To investigate this issue, we challenged a 5HT2 null line of SVG-A cells (B. Assetta, J. Morris-Love, G. V. Gee, A. L. Atkinson, B. A. O'Hara, S. A. Haley, and W. J. Atwood, unpublished data) with purified JCPyV or with EV isolated from JCPyV-infected cells. Infection with purified JCPyV was significantly reduced compared to the level seen with wild-type SVG-A cells, but infection with EV-associated virus was unaffected (Fig. 5). Simian virus 40 (SV40) does not use 5HT2 to infect cells and was therefore used as a specificity control (34–36).

## DISCUSSION

Deep sequencing of viral genomes isolated from the cerebral spinal fluid and brain parenchyma of patients with PML identified a preponderance of genomes with specific mutations in and around the sialic acid (LSTc) binding pocket of the major capsid protein VP1 (13, 14, 37). These mutations render the virus incapable of binding to its major attachment receptor, LSTc, leading some groups to hypothesize the presence of alternative receptors for the virus (13, 14, 37). Geoghegan et al. suggested that JCPyV patient variants may use glycosaminoglycans for infectious entry (38), but those studies focused on JCPyV virus-like particles and pseudovirus variants in the genotype 2 and/or genotype 3 background in a cancer cell line. We have been unable to transduce any of several cell lines in our laboratory with pseudovirus variants from the type 1 genotype. We have, however, shown that these sialic acid binding variants are capable of infecting cells when associated with extracellular vesicles, which could explain the differences in our results.

An analysis of receptor distribution in patient tissues found that both the attachment receptor (LSTc) and the entry receptors (5HT2R) were present on kidney tubules, and virus bound to these tissues in a sialic acid-dependent manner (15). In the same study, the major targets of JCPyV in the CNS, i.e., oligodendrocytes and astrocytes, were devoid of the attachment receptor and failed to bind virus (15). Instead, the attachment receptor was abundantly present on brain microvascular endothelial cells, choroid plexus endothelial (CPE) cells, and microglia, and virus bound to these cells in a sialic acid-dependent manner (15). Subsequent work showed that choroid plexus epithelial cells were fully supportive of JCPyV infection *in vitro*, suggesting that these cells may be a potential driver of JCPyV invasion both in and out of brain parenchyma (16). On the basis of these data and other data showing the critical importance of extracellular vesicles in infection by both enveloped and nonenveloped viruses, we asked whether this could be an alternative mechanism of infection that would explain both the lack of attachment receptor expression on macroglia and the accumulation of virus with critical mutations in the sialic acid binding pocket in patients with PML.

Our data show that JCPyV-infected cells produce abundant extracellular vesicles with JCPyV virions both inside the vesicle and, in some cases, attached to the outside. The vesicles containing virus are much more efficient at infecting cells than purified virus, which is consistent with what others have reported from studies of different viral systems (22). Most importantly, vesicle-mediated infection is not sensitive to neutralization by antiviral antisera and is not dependent on the presence of either the attachment receptor or the entry receptor for the virus. In addition, two of the best-studied sialic acid binding pocket mutants were not capable of transducing SVG-A glial cells as purified virions but readily did so when associated with extracellular vesicles.

These data clarify several major paradoxes in the field and explain how brain cells lacking viral receptors are infected by JCPyV. These data are also the first to demonstrate the use of extracellular vesicles for transmission of any nonenveloped DNA virus, and we suspect that this may well be a universal mechanism for the polyomavirus family. We also suspect that, because choroid plexus epithelial cells are readily infected by JCPyV and produce abundant amounts of extracellular vesicles, these cells may hold the key to neuroinvasion by this common human polyomavirus (39). The ability of virus to evade antibody-mediated neutralization is also likely a critical factor in the pathogenesis of PML.

## MATERIALS AND METHODS

### Cells, viruses, and plasmids.

SVG-A is a transformed cell line derived from primary fetal human glial cells and was described previously (40). SVG-A cells were grown in minimal essential medium (MEM) (Corning, Inc., New York, NY) supplemented with 10% fetal bovine serum (FBS) (Atlanta Biologicals, Flowery Branch, GA) and 1% antifungal/antibiotic (Gibco Life Technologies, Gaithersburg, MD). HEK293FT cells were grown in Dulbecco’s minimal essential medium (DMEM) (Corning) supplemented with 10% fetal bovine serum (FBS), 1% nonessential amino acids (Gibco Life Technologies), and 1% antifungal/antibiotic. EV-depleted medium was used as needed for EV-related experiments, while complete medium was used for general passage of cell lines. EV-depleted medium was produced as described previously (26). Briefly, 2× medium was prepared and spun at 100,000 × *g* in a type 45 Ti rotor (*k*-factor = 133) for 18 h. Medium was then diluted before use to 1× and filtered through a 0.22-µm-pore-size filter (Celltreat, Pepperell, MA). Cells were grown in a humidified chamber at 37°C and 5% CO_2_.

Generation of JC polyomavirus strain Mad-1/SVEΔ was previously described (41, 42). Pseudovirus was produced as described previously (43), with modifications. Briefly, plasmids containing sequences for VP1, VP2, VP3, and CLuc were transfected into HEK293FT cells using FuGENE 6 transfection reagent at a 3:1:1:2 ratio (43, 44). Luciferase reporter vector pSV40_CLuc expresses a secreted form of *Cypridina* luciferase under the control of SV40 early promoter (New England Biolabs, Ipswich, MA). Mock pseudovirus was produced by the same method, substituting nonspecific DNA for the viral expression plasmids. Purification of virus, pseudovirus, and mock pseudovirus was performed as previously described (43, 45).

### Antibodies.

Primary antibodies and the respective dilutions used for Western blotting included annexin V (Abcam, Cambridge, United Kingdom; ab117439) (1:1,000), CD9 (Cell Signaling Technologies, Danvers, MA; CST 13174S) (1:1,000), CD81 (Systems Biosciences, Palo Alto, CA [1:1,000], or BD Biosciences, San Jose, CA [1:2500], or Thermo Fisher Scientific, Waltham, MA [MA5-13548] [1:100]), flotillin-1 (CST 18634S) (1:1,000), calnexin (Santa Cruz Biotechnology, Inc., Dallas, TX; sc-11397) (1:200), cytochrome *c* (BD Pharmingen 556433) (1:500), TSG101 (Thermo PA5-31260) (1:500), and PAB597 (purified) (1:2,000). PAB597 is a monoclonal antibody against VP1 (46). Secondary antibodies used for Western blotting included anti-Mus horseradish peroxidase (HRP) (Thermo A28177) and anti-rabbit HRP (Thermo A27036), both used at 1:10,000. Anti-rabbit 680, anti-mouse 680, anti-rabbit 800, and anti-mouse 800 (Li-Cor, Lincoln, NE) were all used at 1:5,000.

### Purification of extracellular vesicles.

EVs were produced by differential centrifugation, after first pelleting out debris at 300 × *g* in a Sorvall Legend X1R (Thermo) centrifuge for 10 min, and were transferred to a fresh tube and spun at 2,000 × *g* for 10 min. Supernatant was transferred to a fresh tube and spun at 10,000 × *g* in a Sorvall Lynx 6000 (Thermo) centrifuge for 30 min. Supernatant was transferred to Ultra Clear tubes (Beckman Coulter, Brea, CA) and spun at 100,000 × *g* for 70 min in a SW55 Ti rotor (*k*-factor = 139) or 2 h in a SW41 Ti rotor (*k*-factor = 256). The pellet was washed with phosphate-buffered saline (PBS) and repelleted at 100,000 × *g*. The pellet was resuspended in sterile PBS and stored at 4°C for short-term storage or at −20°C for long-term storage. All spins were carried out at 4°C. EV preparations were evaluated by Western blotting, TEM, and nanoparticle tracking analysis (NTA). Generally, EV preparations were characterized and used within 1 week of production.

For negative-staining TEM (Fig. 2C; top 2 panels), EVs were further purified by centrifugation over an OptiPrep gradient (Sigma-Aldrich, St. Louis, MO). Briefly, EVs were layered onto a 27% to 39% stepwise OptiPrep gradient in 5-ml Ultra Clear tubes (Beckman) and spun in a SW55Ti rotor at 237,020 × *g* (*k*-factor = 59) for 3.5 h at 4°C. About 20 fractions were collected and analyzed for density. Fractions were also analyzed by TEM and for infectivity at 3 dpi.

### Infections.

Cells were plated at 10,000 cells/cm^2^ and infected in serum-free media. After infection, inoculum was aspirated and replaced with complete, EV-depleted media. For IFA, cells were fixed at 3 or 5 days postinfection (dpi), before two complete replication cycles occurred. For EV purifications, supernatants were collected between 6 to 8 dpi depending on the health of the cells.

For the antiserum experiment (Fig. 3), virus was pretreated with antisera or preimmune serum at 1:10,000 for 1 h at 4°C. Cells were prechilled at 4°C for 30 min, infected with the pretreated virus on ice, and allowed to bind for 1 h. After infection, cells were washed with PBS and incubated in complete, EV-depleted media containing anti-JCPyV antisera or preimmune serum diluted to 1:10,000. Cells were then shifted to 37°C and then fixed and stained for VP1 at 5 dpi.

### Immunofluorescence analysis.

Cells were fixed and permeabilized in 100% methanol for 30 min at −20°C. After fixation, cells were blocked with 10% goat serum–PBS for 45 min. PAB597 was diluted 1:50 in PBS and incubated on cells for 1.5 h. Cells were washed three times with PBS. Goat anti-mouse secondary antibody conjugated with Alexa-488 Fluor was diluted 1:500 in PBS and incubated on cells for 1 h. Cells were washed three times with PBS and then incubated with DAPI (4′,6-diamidino-2-phenylindole) diluted at 1:1,000 in PBS for 10 min. Cells were washed 2× and imaged on either a Nikon Eclipse TE2000-U or Zeiss Axiovert 200 M fluorescence microscope. The number of DAPI-positive and VP1-positive nuclei per visual field was counted by eye or using Cell Profiler (47).

### Immunoblotting.

Samples were lysed on ice in Pierce radioimmunoprecipitation assay (RIPA) buffer (Thermo) and debris was pelleted out or were lysed in buffer A supplemented with 2.5% deoxycholate (DOC) and 0.01% Triton X followed by 3 freeze-thaws. Both buffers contained cOmplete EDTA-free protease inhibitor cocktail (Roche). Protein content was determined by the use of a Pierce Rapid Gold bicinchoninic acid (BCA) Protein Assay kit (Thermo). Samples were prepared in 4× loading dye (Bio-Rad Laboratories, Hercules, CA), boiled at 95°C for 5 min, and then loaded in 4% to 15% gradient or on 12% Mini-Protean TGX Stain-Free precast gels (Bio-Rad). Stain-Free gels were run at 150 to 200 V and activated and imaged on a ChemiDoc MP imaging system (Bio-Rad) and then transferred to a 0.2-µm-pore-size nitrocellulose membrane by the semidry transfer method. After transfer, Stain-Free gels were reimaged to determine efficiency of protein transfer, and blots were blocked in 1% casein buffer–Tris-buffered saline (TBS) (Bio-Rad). Primary antibodies were diluted in 1% casein and incubated overnight at 4°C. Blots were washed three times with TBS with 0.01% Tween 20 (TBST) and incubated with secondary antibody diluted in TBST for 1 h at room temperature. Horseradish protein (HRP) secondary antibodies were diluted 1:10,000, and fluorescent secondary antibodies were diluted 1:5,000. Blots were washed three times with TBST and then incubated with ClarityMax according to the protocols of the manufacturer (Bio-Rad) for HRP secondary antibodies. Blots were imaged on a ChemiDoc MP imaging system.

### Transmission electron microscopy.

Transmission electron microscopy (TEM) was performed as described previously (18), with modifications. Briefly, 5 µl of sample was adsorbed to a Formvar carbon-coated 400-mesh copper grid for 15 min. The grid was then inverted onto a drop of 1% glutaraldehyde for 1 min and then transferred to fresh drops of distilled water 3 times for 2 min each time. The grid was inverted onto a drop of 3% ammonium molybdate for 2 min to enable the contrast process. The sample was wicked, allowed to dry, and imaged at 100 kV on a Philips 410 transmission electron microscope.

### Immunogold electron microscopy.

Immunogold electron microscopy was performed as described previously (26), with some modifications. Samples were fixed in 2% paraformaldehyde for at least 1 h at 4°C and adsorbed to a Formvar carbon-coated 400 mesh copper grid for 15 min. The grid was washed in PBS 3 times for 3 min each time and placed on drops of 50 mM glycine 4 times for 3 min each time. Grids were blocked in 5% bovine serum albumin (BSA) for 10 min and then incubated with anti-CD81 diluted 1:10 in 1% BSA for 30 min. Grids were then washed in 0.1% BSA 6 times for 3 min each time, followed by washes in 0.5% BSA performed 6 times for 3 min each time. The grids were then incubated on protein A–10-nm gold (Electron Microscopy Sciences, Hatfield, PA) diluted in 0.1% BSA for 20 min. Grids were then washed in PBS 8 times for 2 min each time, followed by fixation in 1% glutaraldehyde for 5 min. Cells were washed with distilled water 8 times for 2 min each time. Samples were contrasted in 3% ammonium molybdate, wicked, dried, and imaged at 100 kV on a Philips 410 transmission electron microscope.

### Neuraminidase treatment.

Type II neuraminidase (Sigma) was used at 0.4 U/ml to treat cells or EV for 1 h at 37°C at pH 6.0. After treatment of EV, the EVs were repelleted at 100,000 × *g* as described above before use in infections.

### Luciferase assay.

Pseudovirus transduction was measured according to the manufacturer’s instructions for an injector-equipped luminometer. Briefly, BioLux *Cypridina* luciferase assay buffer (NEB E3309) was mixed with the CLuc assay solution and then mixed by inversion. The mixture was incubated for 30 min and measured in a Promega Glomax multidetection system with the following parameters: injection of 50 µl of mixture into 50 µl of sample, 2-s delay, and 10-s integration. Samples were read in a white 96-well plate, and luminescence from each sample was measured in triplicate.

### Nanoparticle tracking analysis.

Nanoparticle tracking analysis (NTA) was performed using a Malvern NanoSight NS300 instrument. Samples and standards were diluted in PBS. Nanosphere bead standards (Thermo) were diluted to 1:1,000 and 1:10,000 and used to calibrate the NTA reading, followed by a thorough wash with degassed PBS. After each sample was processed, the NanoSight lines were thoroughly washed. Parameters were set as follows: flow rate of 50 µl/min, with five 30-s videos recorded for analysis.
